# Editorial: CAR T-Cell Therapies in Hematologic Tumors

**DOI:** 10.3389/fonc.2020.588134

**Published:** 2020-10-16

**Authors:** Marta Coscia, Benedetto Bruno, Sattva Neelapu

**Affiliations:** ^1^University Division of Hematology, Azienda Ospedaliero-Universitaria Città della Salute e della Scienza di Torino, and Department of Molecular Biotechnology and Health Sciences, University of Turin, Turin, Italy; ^2^Department of Lymphoma and Myeloma, The University of Texas MD Anderson Cancer Center, Houston, TX, United States

**Keywords:** chimeric antigen receptors, CAR T-Cell therapies, chronic lymphocytic leukemia, B-cell non-Hodgkin lymphoma (B-NHL), multiple myeloma, acute myeloid leukemia (AML), acute lymphoblastic leukemia, CAR T-cell toxicities

## Introduction

Chimeric antigen receptors (CARs) are genetically constructed hybrid receptors that consist of a single-chain variable fragment (scFv) of a monoclonal antibody as the antigen-binding extracellular domain, an intracellular CD3ζ chain as the T cell receptor (TCR) signaling domain, and an additional co-signaling domain, such as CD28 or 4-1BB, to deliver co-stimulation (1, 2). The cytotoxic activity of CAR T cells is determined by antigen-binding to the scFv, which leads to phosphorylation of CD3ζ and additional signaling cascades by co-stimulating domains (3). This mechanism recapitulates intracellular signaling following T-cell activation through the TCR complex, although, in an MHC-independent fashion.

Adoptive T-cell therapy was first clinically employed in 1988 by Rosenberg et al. using *ex vivo* expanded tumor-infiltrating T-cells in melanoma patients (4). At around the same time, the adoptive transfer of CAR-modified T cells was first proposed as an anti-tumor approach (5, 6). Early clinical trials of CAR T cells showed them to be a promising and safe approach, however, therapeutic efficacy was disappointing, likely due to the inability of CAR-modified T cells to expand and persist *in vivo* (7–9). The introduction of co-stimulatory molecules into the signaling domain of CARs greatly increased the potency and persistence of CAR T cells in preclinical studies and their subsequent evaluation in clinical settings demonstrated impressive benefits (10–12). Most successful clinical results have been observed with CD19-directed CAR T cells in B-cell lymphoproliferative disorders (13, 14), which led to the recent FDA and EMA approval of two CAR T-cell therapy products for the treatment of non-Hodgkin lymphomas (NHL) and/or acute lymphoblastic leukemia (ALL). However, in other hematological malignancies, several challenges still need to be overcome for the successful application of CAR T-cell therapies, including identifying target antigens and reversing repressive tumor microenvironments that hamper CAR T-cell function (15, 16).

This collection is comprised of a series of reviews providing a comprehensive overview of the current roles of CAR T-cell therapies in several hematological malignancies while also highlighting challenges for toxicity management and future development.

Greenbaum et al. review the role of CAR T-cell immunotherapy in ALL. They provide an overview of the promising results obtained in clinical trials that led to the approval of anti-CD19 CAR T cells for the treatment of relapsed/refractory pediatric patients with ALL. The authors highlight safety concerns as well as potential reasons that preclude durable responses in some patients as major obstacles to overcome the widespread use of this treatment. They also debate on the role of allogeneic hematopoietic stem cell transplantation as consolidation after CAR T-cell therapy, and present strategies currently under investigation for limiting resistance mechanisms, reducing systemic toxicities, and mitigating obstacles to manufacturing processes.

In their article, Vitale and Strati provide an overview of CAR T-cell therapy in the setting of B-cell NHL and chronic lymphocytic leukemia (CLL). They present results from trials investigating the efficacy and safety of two currently approved second-generation anti-CD19 CAR T-cell products (i.e., axicabtagene ciloleucel and tisagenlecleucel) and a third product (lisocabtagene maraleucel), which is expected to enter the market in 2020. The authors also provide a useful overview of real-world data, including results from special patient populations considered ineligible for pivotal clinical trials (e.g., patients with CNS disease and elderly subjects). Lastly, the review describes novel CAR T-cell-based strategies that are currently under evaluation and which aim to improve efficacy and reduce toxicity in the context of mature B-cell lymphoproliferative disorders.

Lobato et al., review the advances made with CAR T-cell therapy for multiple myeloma. They report results obtained with BCMA-targeted CAR T cells in heavily pretreated patients and outline the potential advantages and disadvantages of new candidate antigens, which are currently under investigation. The authors also provide a thorough description of novel strategies to improve persistence, potency, and trafficking of CAR T cells, such as the refinement of the CAR construct, the use of defined T-cell subpopulations, alternative sources for the production of CAR T cells, the combination approaches with immune modulatory agents, and the use of multi-antigen specific CAR T-cell products.

In their article, Mardiana and Gill provide a comprehensive overview of the current state of CAR T-cell therapy in acute myeloid leukemia (AML). They highlight barriers that limit the full therapeutic potential of this treatment strategy, in particular, the absence of a truly leukemia-specific cell-surface antigen. They also discuss other limitations of CAR T-cell therapy in AML, such as challenges in the manufacturing process due to the presence of leukemic blasts that negatively affect T-cell proliferation and the immunosuppressive mechanisms that diminish CAR T-cell efficacy. The authors also describe potential strategies to overcome such limitations to produce CAR T cells with improved efficacy and tolerability.

CAR T-cell therapies are associated with a unique spectrum of toxicities that are not typically seen with traditional anticancer therapies (17). Sievers et al. in their review focus their attention on these clinical aspects particularly highlighting the role of advanced practice providers (APPs) as members of multidisciplinary care teams in the management of serious adverse events related to these adoptive cell therapies. The article also details approaches for the recognition and grading of the most common CAR T-cell therapy-related toxicities and the available decision support tools for APPs.

In summary, this collection of review articles provides a timely update on the progress made in the field of CAR T-cell therapies for hematologic malignancies, both in terms of the optimal strategies for the management of patients as well as novel products in the development to improve safety and efficacy. Despite the many challenges encountered in moving the field forward, great success has been observed in the last 20 years, leading to the commercial approval of CAR T cells for the treatment of B-cell lymphoproliferative disorders. Many investigations are ongoing to gain a better understanding of the biological mechanisms of toxicity and resistance. These studies on CAR T-cell biology will help develop next-generation strategies to further enhance the efficacy and reduce the toxicity of CAR-transduced cellular therapies, and to extend their applications to other hematological malignancies and solid tumors.

## Author Contributions

All authors listed have made a substantial, direct and intellectual contribution to the work, and approved it for publication.

## Conflict of Interest

MC received Honoraria from Janssen, Gilead, Abbvie, Shire, and research support from Janssen and Karyopharm Therapeutics. BB received Honoraria from Gilead, Pfizer, Thermofisher, Jazz Pharma, Novartis, Janssen, and research support from Janssen. SN served as a Consultant or Advisory Board member for Kite, a Gilead Company, Merck, Bristol-Myers Squibb, Novartis, Celgene, Pfizer, Allogene Therapeutics, Cell Medica/Kuur, Incyte, Precision Biosciences, Legend Biotech, Adicet Bio, Calibr, and Unum Therapeutics; received research support from Kite, a Gilead Company, Bristol-Myers Squibb, Merck, Poseida, Cellectis, Celgene, Karus Therapeutics, Unum Therapeutics, Allogene Therapeutics, Precision Biosciences, and Acerta; received royalties from Takeda Pharmaceuticals, and has intellectual property related to cell therapy.
